# Ultra‐Fast Crystallization of Nanozeolite via Hydroxyl Radicals Generated by Electron‐Beam‐Induced Radiolysis

**DOI:** 10.1002/smsc.202500089

**Published:** 2025-05-04

**Authors:** Charles Sidhoum, Abdallah Amedlous, Clément Sanchez, Ovidiu Ersen, Svetlana Mintova

**Affiliations:** ^1^ Laboratoire de Chimie de Matière Condensée Collège de France Sorbonne Université 75005 Paris France; ^2^ Institut de Physique et Chimie des Matériaux de Strasbourg CNRS Université de Strasbourg 67037 Strasbourg France; ^3^ ENSICAEN CNRS LCS Université de Caen Normandie 14000 Caen France; ^4^ University of Strasbourg Institute for Advanced Study (USIAS) Université de Strasbourg 67000 Strasbourg France

**Keywords:** fast crystallization, nanozeolites, nucleation and growth, radiolysis, transmission electron microscopy

## Abstract

The development of nanozeolites is crucial for advancing applications in catalysis, adsorption, and separation due to their unique structural and functional properties. Herein, we demonstrate the ultra‐fast crystallization of RHO‐type nanozeolite (in just a few tenths of a seconds) in a colloidal aluminosilicate suspension is demonstrated and is observed by transmission electron microscopy. Nucleation occurs almost instantaneously, within 5 s, under electron beam irradiation, followed by rapid, homogeneous crystal growth completed within 14–20 s. The crystallization of RHO nanocrystals is driven by hydroxyl radicals (OH^•^) generated through radiolysis, allowing for real‐time tracking of nanozeolite formation. By systematically varying the electron dose rate from 0.66 to 80.7 e^−^ Å^−2^ s^−1^, its critical role in controlling induction time, nucleation density, and particle coalescence is demonstrated. The latter stages involve Ostwald ripening, resulting in the formation of larger RHO nanocrystals. Notably, coalescence occurs earlier at higher electron doses (80.7 e^−^ Å^−2^ s^−1^) due to accelerated nucleation from a higher generation of OH^•^ radicals. These findings provide direct evidence of the ultra‐fast kinetics of nanozeolite nucleation and growth, highlighting the pivotal role of hydroxyl radicals in driving amorphous nanoparticle formation and stabilizing zeolite crystallites with uniform crystals size.

## Graphical abstract

Electron beam‐induced hydroxyl radicals (OH^•^) trigger rapid nucleation and ultra‐fast crystallization of RHO nanosized zeolite, as revealed by high‐resolution transmission electron microscopy. The findings highlight the key role of OH^•^ radicals in initiating the formation of amorphous nanoparticles and stabilizing uniformly sized zeolite crystals in colloidal suspensions.

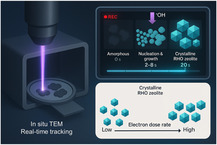

## 1 Introduction

Zeolites are a class of microporous crystalline aluminosilicates that play a crucial role in industrial processes including gas separation, ion exchange, and catalysis.^[^ ^1^ ^]^ Their chemical compatibility, thermal stability, versatility, and low cost have made them one of the most widely used catalysts in petrochemistry,^[^ ^2^ ^]^ biomass conversion,^[^ ^3^ ^]^ and absorption processes.^[^ ^4^ ^]^ Over the past decades, significant efforts have been made to develop new synthesis approaches and to establish the link between structure and properties in this class of materials. In particular, recent developments in the synthesis of nanosized zeolites have extended this already broad field of research and opened the door to novel applications. Nanozeolites offer several advantages over their conventional microsized counterparts, such as reduced diffusion lengths, minimized mass transport limitations, and the ability to process molecules larger than the zeolite pores, primarily on the external surface. These properties help prevent unwanted secondary reactions and coke formation while ensuring high stability at elevated temperatures and in colloidal suspensions.^[^ ^5^ ^]^ Although considerable efforts have been made recently to develop their synthesis, the mechanisms behind their formation remain uncertain, opening room to a whole new field of exploration.^[^ ^6^, ^7^ ^]^

The classical pathway of zeolite synthesis involves an alkaline medium and a hydrothermal‐based approach. The typical aluminosilicate network is first obtained as an amorphous gel formed by polymerization. A second depolymerization step breaks the aluminosilicate network to obtain aluminosilicates and silicates in suspensions in the case of dense gels. The last step involves the repolymerization of silicate species, forming Al,Si—O—Si bonds through polycondensation reactions. This three‐step crystallization process is time consuming, taking hours or even days, depending on the chemical and physical parameters of the synthesis. High‐temperature synthesis is typically employed to accelerate kinetics by increasing the reaction rate, but this involves high‐energy consumption.^[^ ^8^ ^]^ However, many efforts have been made in recent years to speed up the crystallization with original synthetic approaches.^[^ ^9^ ^]^ A systematic control on the initial parameters (chemical composition, quantity, and size of the seeds)^[^ ^10^ ^]^ as well as the use of organic structure directing agents^[^ ^11^, ^12^ ^]^ can significantly accelerate the kinetics of crystallization. Other methods such as continuous‐flow synthesis^[^ ^10^ ^]^ or interzeolites transformation^[^ ^7^, ^13^ ^]^ can be used with potential industrial interests. The role of radicals has also been explored in few works detailed in the following paragraphs.

The synthesis of zeolites generally occurs in an alkaline medium, which is justified by the crucial role of successive Si—O bond breaking and reforming, with OH^−^ ions acting as catalysts.^[^ ^14^ ^]^ Theoretical calculations have shown that the use of hydroxyl radicals (OH^•^) can lower the energy barrier of the Si—O rupture compared to the same phenomenon catalyzed by the hydroxide ions.^[^ ^15^ ^]^ Recent experimental studies support this findingu, demonstrating a considerable increase in the kinetics of zeolite crystallization with low energy consumption, thereby raising tremendous expectations for industrial applications.^[^ ^14^ ^]^ Feng et al. combined electron paramagnetic resonance (EPR) with theoretical calculations to study the influence of OH^•^ radicals generated by UV irradiation or by the addition of Fenton's reagent under hydrothermal conditions.^[^ ^16^ ^]^ Their results indicate that OH^•^ mostly influences the nucleation stages, considerably accelerating the crystallization kinetics, a finding further confirmed by other works from the same group.^[^ ^17^, ^18^ ^]^ Chen et al. used gamma irradiation to induce the formation of OH^•^ radicals and reduced the synthesis time from 102 to 18 h without adding any supplementary reagents.^[^ ^19^ ^]^ The use of other physical methods (plasma technology, water sonolysis) can also generate OH^•^ radicals and speed up the kinetics, with EPR analysis remaining the primary detection method.^[^ ^20^ ^]^

Interestingly, high‐energy electron beam irradiation is well‐known to generate many effects on materials including the radiolysis of molecules.^[^ ^21^ ^]^ Indeed, in liquid‐phase transmission electron microscopy (LP‐TEM), exposing a liquid to the beam induces its radiolysis, leading to the formation of radiolytic species. For example, in the case of water molecules, the electron beam causes their decomposition and the generation of different species, known as primary products of water radiolysis (Equation (1)):^[^ ^22^ ^]^

(1)

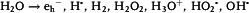




The radiolysis effect is a central aspect of any LP‐TEM experiment and is generally considered detrimental due to the damage it causes to the sample. However, a few studies have taken advantage of this effect in a beneficial manner for the synthesis of metallic materials.^[^ ^23^, ^24^, ^25^ ^]^ In fact, the development of irradiation‐assisted synthesis, not only with an electron beam, is a promising field of research^[^ ^26^ ^]^ and has also started to explored zeolites crystallization but without a mechanistic point of view.^[^ ^27^ ^]^

Here, we report on the generation of OH^•^ radicals by radiolysis of water molecules, which induces the crystallization of nanosized RHO zeolites by in situ TEM. Using a highly supersaturated colloidal suspension, no supplementary reagents (e.g., Fenton reagent or persulfate‐based agents) or high‐temperature treatments are required to initiate the crystallization of highly homogeneous RHO zeolite nanoparticles. Furthermore, the crystallization of the nanozeolites is achieved in an ultra‐short time in just a few tenths of a seconds, demonstrating the significant impact of hydroxyl radicals on the kinetics of the crystallization process. More particularly, this work provides direct monitoring of the crystallization of nanosized RHO‐type zeolite under the electron beam. While it is possible to conduct experiments in a liquid microchip‐cell using LP‐TEM, our findings indicate that the substantial water content in the colloidal suspension is sufficient to generate radiolytic species without the need for specialized setups. Moreover, the suspension remains stable under the vacuum conditions of the TEM column.

## 2 Results and Discussion

To quantitatively assess the effect of electron beam irradiation on the crystallization process, we evaluated the energy received by the material, expressed as a dose in grays (Gy) or as a dose rate when reduced to a time unit (e.g., Gy/s). The electron dose and electron dose rate are usually expressed by the number of electrons per unit of sample irradiated, respectively, in e^−^ Å^−2^ and e^−^ Å^−2^ s^−1^ (see TEM section in Supporting Information). Note that the cumulative electron dose, also expressed in e^−^ Å^−2^, can be calculated by multiplying the dose rate by the irradiation time during which the sample was exposed. The synthesis of RHO nanozeolite was conducted at various electron dose rates and cumulative electron doses to investigate their effects on the crystallization process.

The colloidal suspension studied here, referred to as nano‐RHO has the following chemical composition: 10 SiO_2_:0.8 Al_2_O_3_:8 Na_2_O:0.58 Cs_2_O:100 H_2_O.^[^ ^28^ ^]^ For further details, please refer to the synthesis section in the Supporting Information.

To probe the evolution of the suspension under conventional hydrothermal treatment conditions and compare it with subsequent *in situ* TEM observations, the transformation of the amorphous suspension into crystalline RHO‐type zeolite was monitored using Raman spectroscopy, supported by X‐ray diffraction (XRD) analysis at various stages of crystallization (Figure S1a,b, Supporting Information). During the initial stages of hydrothermal treatment (0–10 min), Raman spectroscopy revealed a weak, broad peak at 480 cm^−1^, characteristic of amorphous matter containing four‐membered rings (4‐MR). After 10 min, significant changes were observed, with the emergence of sharper peaks at 521 cm^−1^ (4‐MR in crystalline materials^[^ ^29^, ^30^ ^]^), 426 cm^−1^ (6‐MR), and 266 cm^−1^ (8‐MR), indicating the onset of crystallization and the formation of an RHO‐type structure.^[^ ^31^, ^32^, ^33^ ^]^ This suggests that aluminosilicate species in the colloidal precursor suspension exhibit a short‐range crystalline arrangement, detectable by Raman spectroscopy well before the appearance of long‐range crystalline features in the XRD analysis. While Raman detected the development of crystalline structures as early as 10 min, XRD only revealed the first weak Bragg peaks associated with RHO zeolite after 30 min, and the crystallization was completed after 40 min. Thus, Raman spectroscopy proves more sensitive for detecting early‐stage crystallization than XRD in this specific process. However, these results demonstrated the formation of RHO zeolites within tens of minutes and the presence of 4‐MR rings in the amorphous suspension. These low‐coordinated structures (4‐MR rings) have already been studied to explain the different nature of species in colloidal silica suspension and fumed silica.^[^ ^34^ ^]^ The increased presence of low‐coordinated rings in fumed silicas, as confirmed by EPR analyses,^[^ ^28^ ^]^ may help explain the influence of hydroxyl groups on the kinetics of amorphous structures containing 4‐MR rings.

To monitor the influence of the radiolytic species generated by the electron beam on the kinetics of RHO zeolite crystallization, we deposited the suspension used for the study above onto a standard carbon coated copper grid for TEM analysis. Initially, we demonstrated that the suspension remains stable under very low electron dose rates (0.22 e^−^ Å^−2^ s^−1^, Figure S4, Supporting Information) without any significant changes, even after 20 min of irradiation, corresponding to a cumulative electron dose of 283.5 e^−^ Å^−2^. When the electron dose was slightly increased to 0.66 e^−^ Å^−2^ s^−1^ (Figure S5, Supporting Information), minor electron beam effects were noticed after 20 min (cumulative electron dose of 793.2 e^−^ Å^−2^), resulting in slight degradation of the aluminosilicate matrix and the appearance of clearer areas (Figure S5c, Supporting Information). At an electron dose of 0.91 e^−^ Å^−2^ s^−1^ (Figure S6, Supporting Information), these effects were more pronounced, appearing within 10 min of exposure, accompanied by noticeable shrinkage of the suspension (Figure S6c, Supporting Information). The cumulative dose used to monitor the changes in the suspension in Figure S6c, Supporting Information (546 e^−^ Å^−2^) was lower than that in Figure S5c, Supporting Information, yet the observed effects were more significant, particularly the shrinkage of the aluminosilicate suspension. This suggests that the electron dose rate plays a more critical role in affecting the system than the total cumulative electron dose alone. However, no crystallization and particles formation are observed at low electron doses (<1 e^−^ Å^−2^ s^−1^), even over extended exposure times.

**Figure 1:**
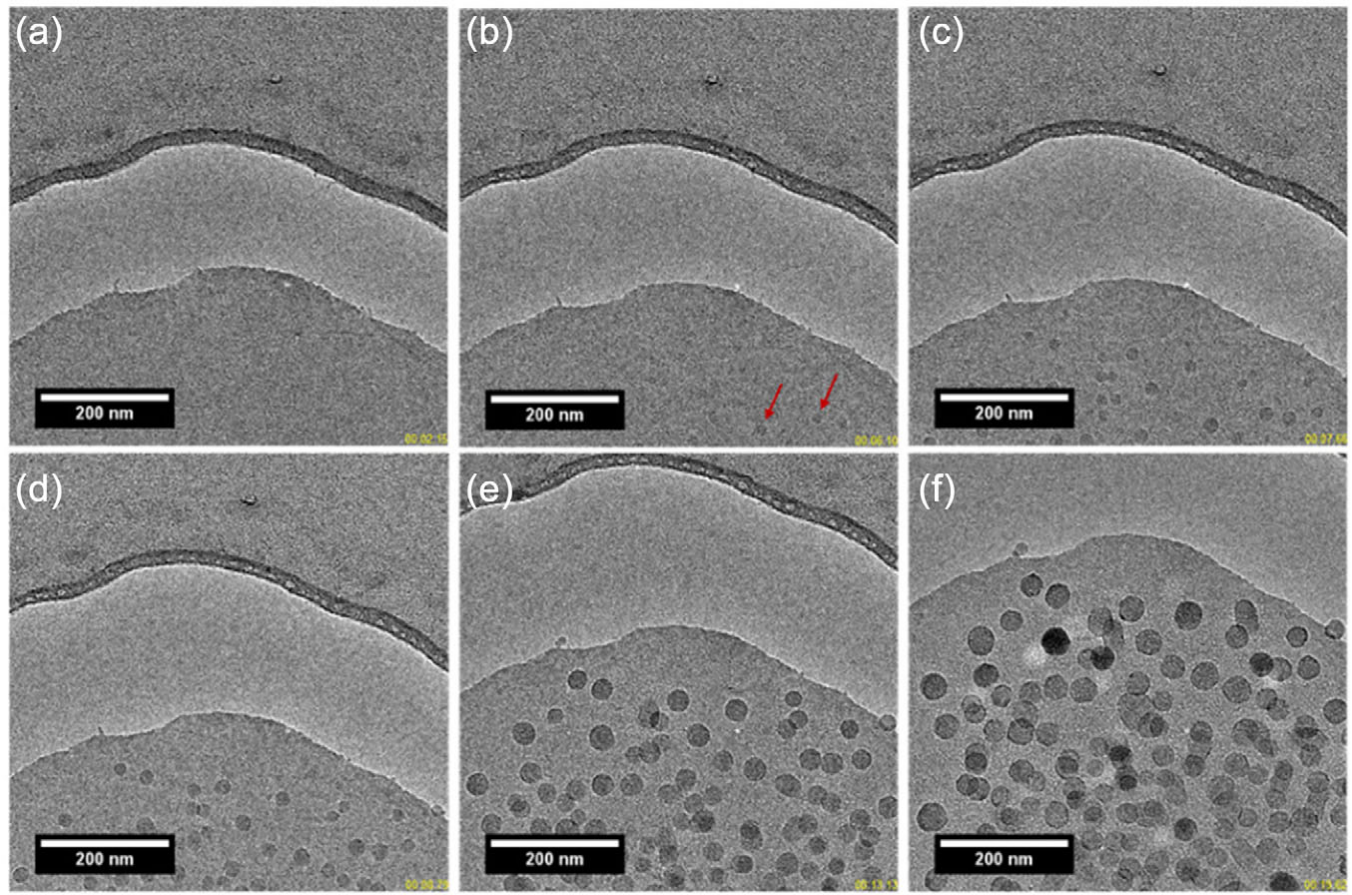
TEM images sequence showing the growth of nanosized RHO zeolite crystals within the aluminosilicate suspension (electron dose = 61.3 e^−^Å^−2^ s^−1^). a) 0.6 s, b) 5.6 s, c) 7.6 s, d) 9.27 s, e) 14.93 s, and f) 23.6 s. The red arrows indicate the initial center of nucleation. See also Movie S2, Supporting Information.

The influence of the electron dose rate as a critical parameter was confirmed when it was significantly increased. At an electron dose rate of 24.6 e^−^ Å^−2^ s^−1^ (Figure S7 and Movie S1, Supporting Information), rapid nucleation and growth of particles with a size of several tens of nanometers, were observed. In fact, even in the first acquired image (Figure S7a, Supporting Information), large contrasting particles were visible within the aluminosilicate suspension at a cumulative electron dose of 246 e^−^ Å^−2^ s^−1^, which was four times lower than that used for monitoring the changes in the suspension presented in Figure S5c, Supporting Information. This confirmed that the electron dose rate is the primary factor initiating nucleation and growth of nanozeolites. Therefore, this parameter will be the focus of the subsequent study. The particles formed initially continued to grow over time (Figure S7b–d, Supporting Information) through successive coalescence and dissolution processes of smaller aluminosilicates, akin to Ostwald ripening growth (see Movie S1, Supporting Information). They reached sizes in the hundreds of nanometers after a few minutes, and a change in morphology indicated degradation under prolonged electron beam exposure (Figure S7e, Supporting Information). Due to the rapid formation, early‐stage information was difficult to capture. To address this, we implemented a “blank procedure” that allowed us to record the initial stage of irradiation (see Supporting Information). Using this approach, we successfully captured the nucleation and growth of nanosized RHO crystals induced by electron beam in the colloidal aluminosilicate suspension. **Figure** **1** presents direct monitoring of the nucleation and growth of RHO nanozeolites from the suspension at an electron dose rate of 61.3 e^−^ Å^−2^ s^−1^, noted d_e1_ (see also Movie S2, Supporting Information).

After just a few seconds, zeolite nucleation begins (Figure 1b), followed by rapid growth over ≈20 s (Figure 1c–f). The ultra‐fast kinetics of RHO nucleation and growth using an electron dose of 61.3 e^−^ Å^−2^ s^−1^ is observed here in comparison to the slow kinetics in suspension subjected to hydrothermal treatment, as revealed by Raman and XRD analysis (Figure S1a,b, Supporting Information). This observation is consistent with the radiolysis of the water molecules within the suspension, leading to the generation of hydroxyl radicals that have a significant influence on the nucleation and growth of zeolite crystals. Interestingly, the zeolite crystals exhibit homogeneous growth despite the inherently unpredictable nature of radiolysis within the colloidal aluminosilicate suspension. We hypothesize that at this relatively high electron dose rate, a substantial amount of radical species is produced, generating numerous nucleation centers and enabling uniform particle growth. This interpretation aligns with previous work on different systems.^[^ ^26^ ^]^ Additionally, several particles displayed faceted morphologies and diffraction contrast during the movie recording (Movie S2, Supporting Information), further suggesting the formation of crystalline particles.

**Figure 2:**
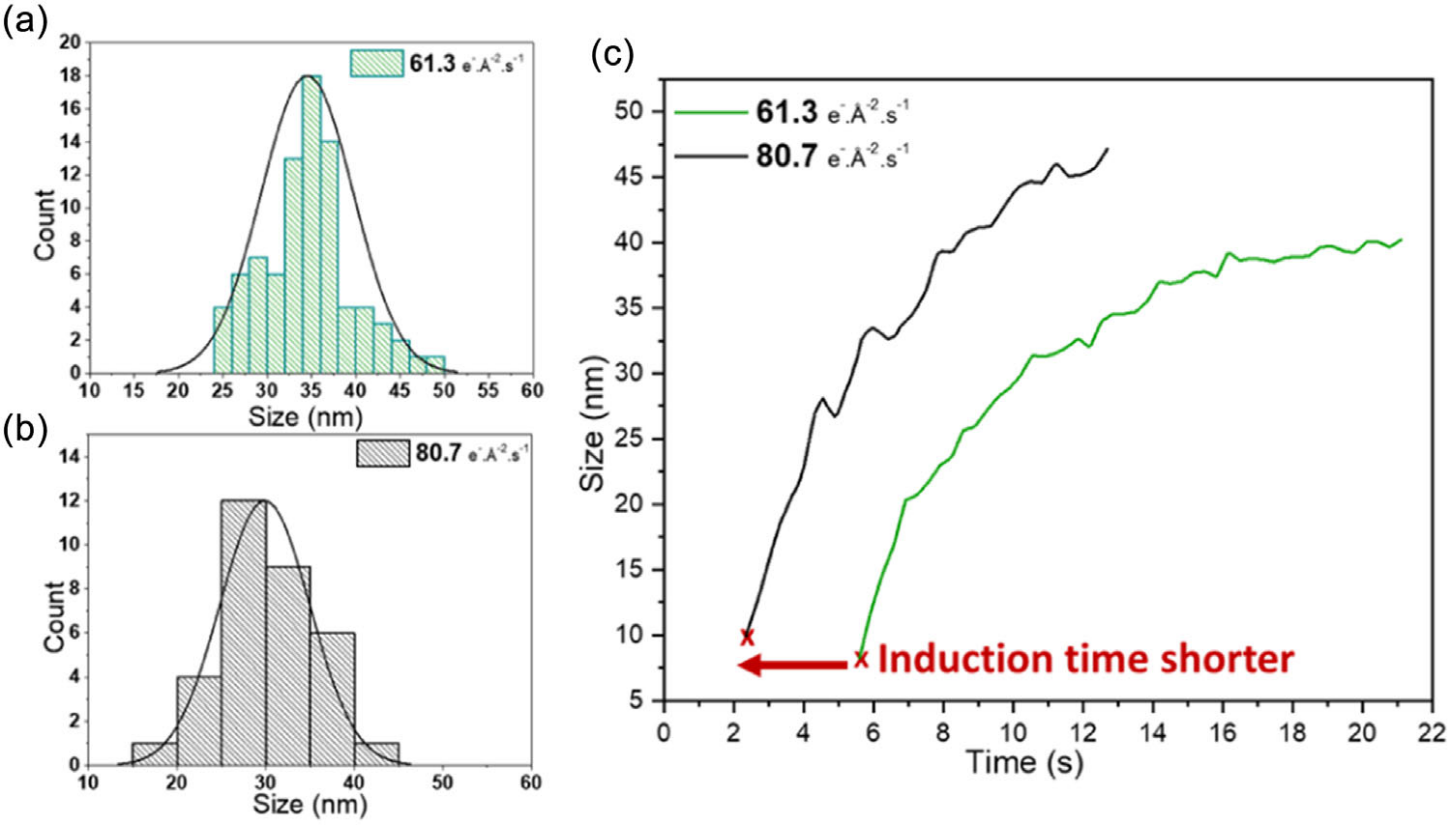
Size distribution of the nanosized zeolite crystals obtained at an electron dose rate of a) 61.3 e^−^ Å^−2^ s^−1^; b) 80.7 e^−^ Å^−2^ s^−1^; and c) average particle size evolution over time, measured based on the results presented in Figure S9a,c, Supporting Information, at two different electron dose rates. Note that the particles that have already coalesced are not considered for the size distribution presentation. The red arrow indicates the difference in induction times at an electron dose rate of 61.3 e^−^ Å^−2^ s^−1^ (green) and 80.7 e^−^ Å^−2^ s^−1^ (black).

We performed another recording at an electron dose rate of 80.7 e^−^ Å^−2^ s^−1^, labeled d_e2_, (Figure S8 and Movie S3, Supporting Information), which showed a similar process of nucleation and homogeneous crystal growth within 10 s. The average size of the two populations observed at the end of the growth and before coalescence of multiple particles for d_e1_ and d_e2_ was measured using the images presented in Figure 1f and Figure S8f, Supporting Information. The size distribution histograms (**Figure** **2**a,b) indicated an average particle size of 34 ± 5 nm and 28 ± 5 nm, respectively, for the samples synthesized and denoted as d_e1_ and d_e2_. Note that similar average sizes of RHO crystals were obtained using both electron dose rates, indicating the presence of a critical radius before particle coalescence. However, coalescence occured earlier for d_e2_, which is certainly due to faster nucleation, i.e., shorter induction time, accelerated by the higher amount of OH^•^ radicals generated by radiolysis. This observation supports previous findings by Feng et al. which suggest that radicals influence the nucleation stage, but not the crystal growth stage.^[^ ^16^ ^]^

To further investigate the kinetics of crystallization before coalescence, we measured the size evolution of three distinct particles at the electron dose rate of d_e1_ and d_e2_, as presented in Figure S9a,b and S6c,d, Supporting Information, respectively. The change of crystal size as a function of time for each electron dose rate is shown in Figure 2c, highlighting a clear difference in induction time, thus supporting the hypothesis of faster nucleation of RHO zeolite at high electron doses. However, in both cases, the crystallization process follows the same pathway, i.e., a similar growth mechanism involving the formation of solid aluminosilicate particles in aqueous suspensions and their transformation into RHO nanocrystals with homogeneous sizes.

**Figure 3:**
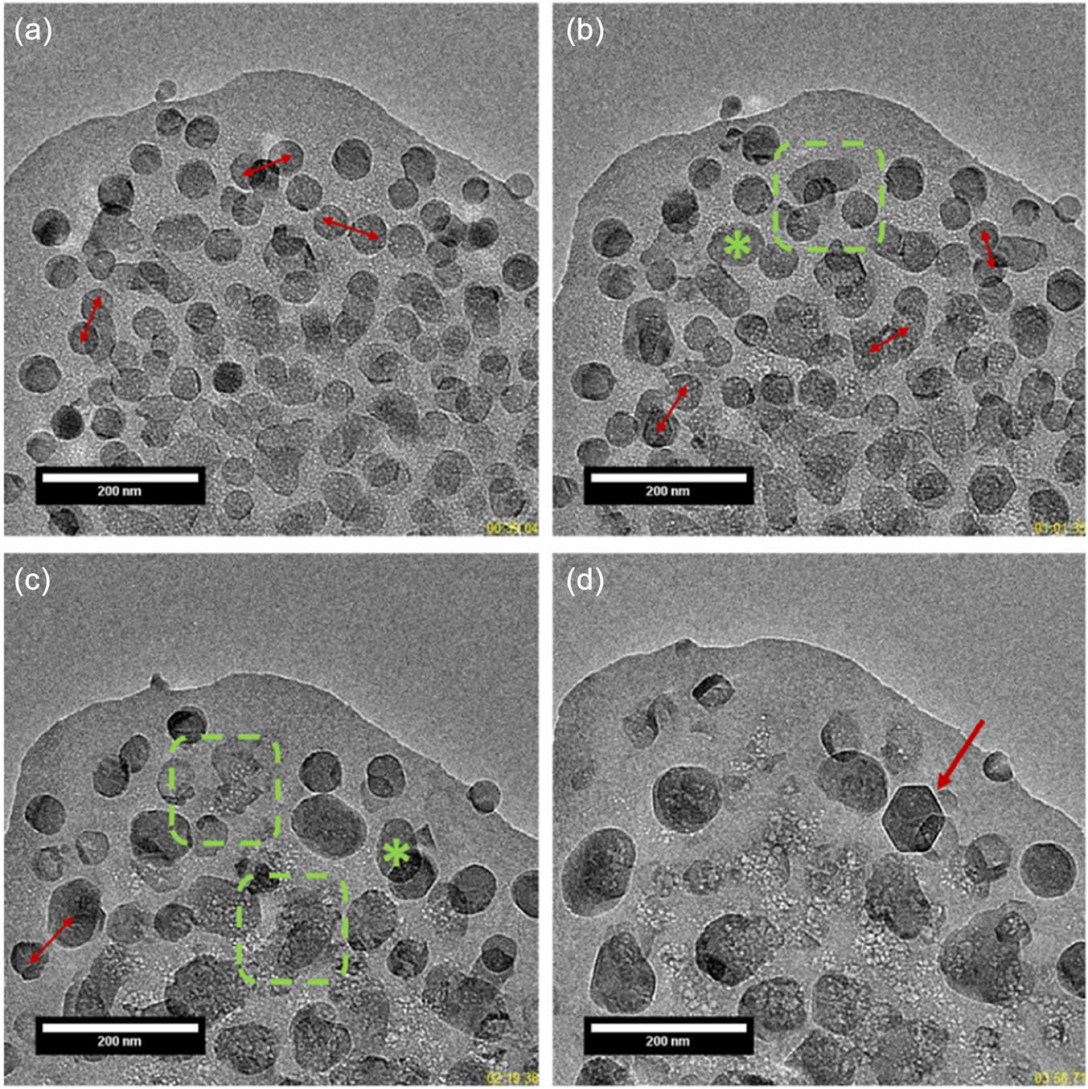
TEM images at different time intervals after the initial homogeneous growth of RHO nanosized zeolite: a) 36.63 s, b) 58.94 s, c) 146.97 s, and d) 236.3 s. Electron dose rate: 61. 3 e^−^ Å^−2^ s^−1^. Double red arrows indicate the coalescence of two particles; green dotted squares mark the area that dissolved during the Ostwald ripening; green star indicates a particle growing during the Ostwald ripening; red single arrow denotes large, faceted RHO particle observed at the end of the crystallization process. See also Movie S4, Supporting Information.

As previously mentioned, particle coalescence was observed, followed by Ostwald ripening. **Figure** **3** displays an example of the different events occurring after the initial growth (see also Movie S4, Supporting Information) for the dose d_e1_. Note that these events are reproducible and similar across the different dose rates studied (see Movie S1 and S5, Supporting Information). We finally observed very large, sometimes well‐faceted particles(Figure 3d), with sizes on the order of hundreds of nanometers. We were also able to image the Ostwald ripening phenomenon at high resolution, providing a direct view with excellent spatial resolution of this important step (Figure S10 and Movie S6, Supporting Information).

## 3 Conclusion

**Figure 4:**
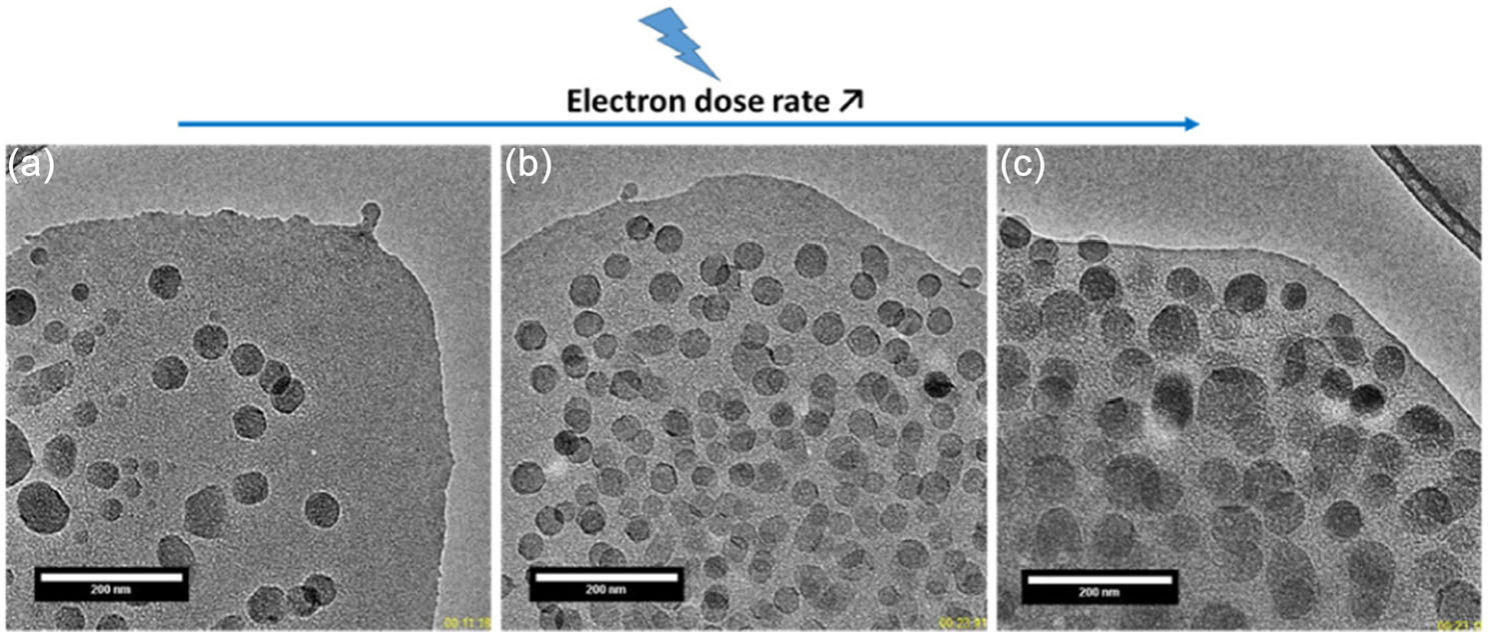
TEM images captured at different electron dose rates after a similar irradiation time of 20 s, illustrating the effect of increasing the electron dose rate on the kinetic and generation of nucleation centers and growth of RHO nanosized zeolite. a) 24.6 e^−^ Å^−2^ s^−1^, b) 61.3 e^−^ Å^−2^ s^−1^, and c) 80.7 e^−^ Å^−2^ s^−1^.

In summary, we directly monitored the crystallization of nanosized RHO zeolite assisted by the generation of radiolytic hydroxyl radicals induced by the electron beam. A comparison of the crystal growth kinetics of nanosized RHO zeolites under conventional hydrothermal treatment conditions and in situ TEM, reproducible across different electron doses, demonstrated the significant impact of these radicals on the crystallization process, particularly during the nucleation stage (**Figure** **4**). After an initial phase of individual particle growth, the particles underwent coalescence, and larger particles formed via Ostwald ripening.

These findings align with recent studies on driving nanomaterial crystallization by irradiation,^[^ ^26^ ^]^ opening up new synthetic avenues for producing nanozeolites and offering crucial insights into their nucleation and growth mechanisms. Furthermore, with the recent advances in quantitative simulation of radiolysis products generated by the electron beam,^[^ ^25^, ^35^ ^]^ we can expect this type of work to lay the foundations for quantitative radiolysis studies using TEM. From a more general point of view, this study opens a land of opportunities for tailoring inorganic or hybrid nanomaterials through a radical‐driven chimie‐douce pathway.

## Conflict of Interest

The authors declare no conflict of interest.

## Author Contributions

**Charles Sidhoum**: data curation (lead); formal analysis (lead); investigation (lead); writing—original draft (lead). **Abdallah Amedlous**: data curation (equal); formal analysis (equal); investigation (equal). **Clément Sanchez**: conceptualization (lead); supervision (lead). **Ovidiu Ersen**: conceptualization (equal); resources (equal); supervision (equal); writing—review & editing (equal). **Svetlana Mintova**: funding acquisition (equal); resources (equal); supervision (equal); writing—review & editing (equal).

## Supporting information

Supplementary Material

## Data Availability

The data that support the findings of this study are available in the Supporting Information of this article.
