# Supplementary material for: Ultra‐Fast Crystallization of Nanozeolite via Hydroxyl Radicals Generated by Electron‐Beam‐Induced Radiolysis
Source: Small Sci. 2025 May 4;5(7):2500089. doi: 10.1002/smsc.202500089 (PMC12257879; doi:10.1002/smsc.202500089)
Supplement: Supplementary file 1 — Supplementary Material [file SMSC-5-2500089-s001.zip › Supporting Information/smsc202500089-sup-0001-SuppData-S1.pdf]

Supporting Information

Ultra-Fast Crystallization of Nanozeolite via Hydroxyl Radicals Generated by Electron-Beam Induced Radiolysis

Charles Sidhoum,[a-b] Abdallah Amedlous,[c] Clément Sanchez,[a-d] Ovidiu Ersen,*[b] and Svetlana Mintova*[c]

[a] Dr. C. Sidhoum, Prof. Dr. Clément Sanchez
Laboratoire de Chimie de Matière Condensée
Sorbonne Université, Collège de France
75005, Paris, France

[b] Dr. C. Sidhoum, Prof. Dr. Ovidiu Ersen
Institut de Physique et de Chimie des Matériaux de Strasbourg
CNRS, Université de Strasbourg
67037, Strasbourg, France
E-mail : ovidiu.ersen@ipcms.unistra.fr

[c] Dr. Abdallah Amedlous, Dr. Svetlana Mintova
Université de Caen Normandie, ENSICAEN, CNRS, LCS, 14000 Caen, France
E-mail : svetlana.mintova@ensicaen.fr

[d] Prof. Dr. Clément Sanchez
University of Strasbourg Institute for Advanced Study (USIAS)
Université de Strasbourg
67000, Strasbourg, France

Experimental Section

Chemical and Materials

Sodium aluminate (53% Al_2O_3 47% Na_2O wt.%), colloidal silica (Ludox AS40, 40 wt.% of SiO_2), and sodium hydroxide pellets (NaOH, 98%), were purchased from Sigma-Aldrich. Cesium hydroxide hydrate ($CsOH.H_2O$, 99.99%), was purchased from Alfa Aesar. All reagents were used as received without further purification.

Preparation of colloidal suspension for TEM characterization

In a typical synthesis of nanosized RHO type zeolite, a precursor suspension with the molar composition 10 SiO_2 : 0.8 Al_2O_3 : 8 Na_2O : 0.58 Cs_2O : 100 H_2O was prepared. Initially, 0.51 g of sodium aluminate ($NaAlO_2$) was dissolved in 3 g of deionized water (H_2O) to form a clear solution. To this solution, 1.82 g of sodium hydroxide (NaOH) and 0.58 g of cesium hydroxide (CsOH.H2O) were added. The mixture was stirred vigorously at room temperature (25°C) for 2 hours, resulting in a clear colloidal suspension. Subsequently, 5 g of LUDOX AS40 (colloidal silica, SiO_2) was added dropwise under continuous stirring at room temperature. This led to the formation of the precursor suspension, which was then used for further experiments.

Ex situ synthesis of RHO zeolite

For the ex situ synthesis of RHO zeolite, the precursor suspension was subjected to hydrothermal treatment in 60 mL propylene bottles at 90 °C for different times of crystallization (0-60 min). The same initial precursor suspension was used for both the in situ TEM studies and the ex situ synthesis.

Ex-situ characterizations of amorphous and crystalline RHO zeolite nanoparticles

Powder X-ray diffraction (PXRD) patterns were collected with a PANalytical X'Pert Pro diffractometer using Cu Kα1 radiation (λ = 1.5406 Å, 45 kV, 40 mA). The patterns were collected between 5–60° 2θ with a step size of 0.0167 and time per step of 1000 s.

The average size of particles in the amorphous and crystalline suspensions was determined by dynamic light scattering (DLS) using a Malvern Zetasizer Nano instrument.

The *Ex-situ FTIR* spectra of samples were acquired on a Nicolet Nexus FTIR spectrophotometer for 128 scans with a 4 cm^{-1} optical resolution using KBr as a reference. Self-supported wafers with a diameter of 1.6 cm were diluted with KBr (1% sample, 99% KBr) and hydraulically pressed (15 mg/cm^2, Surface area = 2 cm^2).

Raman spectra were collected on a Horiba Jobin Yvon, HR Evolution spectrometer equipped with a microscope (100× objective) and a CCD detector. A 532 nm wavelength laser was used, with each spectrum accumulated four times for 30 seconds.

Transmission Electron Microscopy

Imaging, chemical analysis and electron diffraction were obtained with a corrected JEOL 2100F/Cs Transmission Electron Microscope (TEM) operating at 200kV. Samples were deposited on a holey carbon-coated TEM grid and characterized directly in the ultra-high vacuum conditions of the TEM column. Chemical analysis using Energy-Dispersive XRay Spectroscopy (EDXS) were obtained using a SDD detector. For preliminary analysis (Figure S3-7), imaging was performed using a LaB_6 JEOL 2100 operating at 200kV.

In order to observe the very first moments of nucleation and growth of the particles, a “blank procedure” was performed. The beam was blanked and the sample displace randomly. The video acquisition was started before stopping the beam blank. Hence the very first image of each video corresponds to the first moment of irradiation. A time offset corresponding the few seconds of acquisition without the beam on was applied to each kinetic curve for considering the real t_0.

To estimate the electron dose, the brightness of the electron beam was adjusted in TEM mode to illuminated the entire phosphorous screen. The beam current was read on the JEOL software in pA/cm² before being converted into $e^-.Å^{-2}.s^{-1}$ taking into account the magnification. The electron dose was varied by changing the beam spot size at constant magnification.

Note that for the images obtained in Figure S.3-6 an approximate time of 30sec of illumination was considered for the first image (t_0) to evaluate the cumulative dose. Regarding the absence of significative effect of the beam for shorter times on these acquisitions, this does not lead to any misinterpretation.

Study of crystal growth of nanosized RHO zeolite by ex-situ Raman spectroscopy and X-ray diffraction

The transformation of an amorphous aluminosilicate precursor into a crystalline RHO-type zeolite was monitored using Raman spectroscopy, supported by X-ray diffraction (XRD) analysis at various stages of crystallization. Samples were taken at specific time intervals during the synthesis.

The Raman spectra of samples at the initial stages of hydrothermal treatment (0–10 minutes) (Figure S 1a) contain a weak, broad peak at 480 cm^{-1}, characteristic of amorphous matter containing four-membered rings (4-MR). XRD analysis (Figure S 1b) confirmed this observation, showing that the precursor remained fully amorphous up to 20 minutes of heating. After 10 minutes, significant changes were observed in the Raman spectra. A sharper, more intense peak emerged at 521 cm^{-1}, alongside new peaks at 426 cm^{-1} and 266 cm^{-1} appeared. The peak at 521 cm^{-1} is attributed to the breathing vibration mode of four-membered rings (4-MR) in crystalline matter, while the peaks at 426 cm^{-1} and 266 cm^{-1} correspond to six-membered rings (6-MR) and eight-membered rings (8-MR), respectively. These results suggest the formation of a short-range crystalline order within the amorphous precursor that correspond to the RHO-type structure after 10 minutes. The shift of the initial peak at 480 cm^{-1} to 521 cm^{-1}, along with increased intensity, signifies the onset of crystallization, suggesting that aluminosilicate species in the precursor begin to connect into a long-range crystalline structure. The Raman results indicate that short-range crystalline arrangement in the aluminosilicate precursors occurs after 10 minutes, while a fully crystalline RHO zeolite is formed after 40 minutes. However, the appearance of crystalline features detectable by XRD is only observed after 30 minutes of hydrothermal treatment. The first Bragg peaks, with very low intensity corresponding to the RHO zeolite appeared in the sample treated for 30 minutes and they increased further with the time of hydrothermal treatment (Figure S 1b). In summary, the formation of the RHO zeolite at very early stages of hydrothermal treatment is detected by Raman spectroscopy much earlier than by XRD.

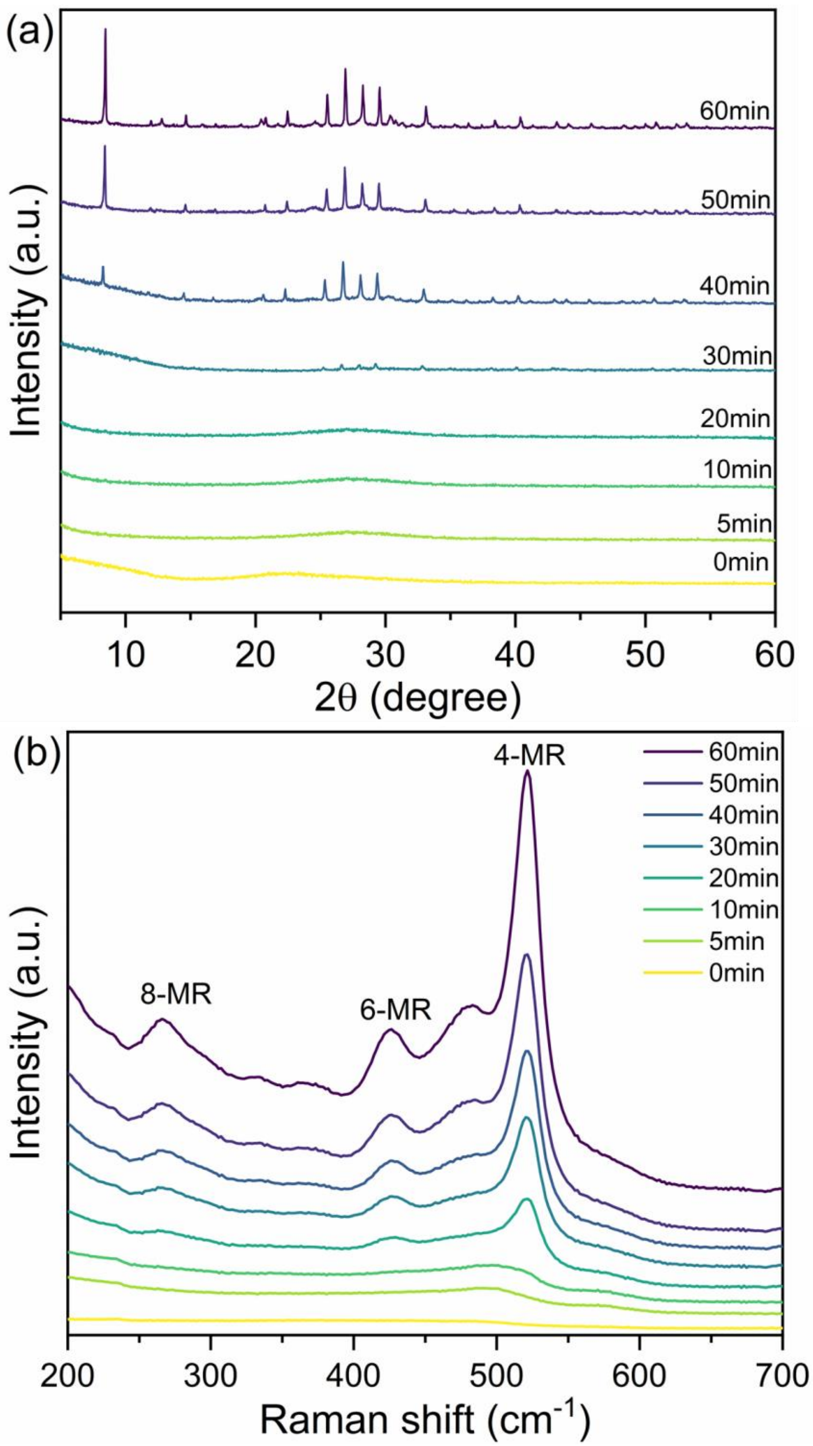

Figure S 1: Raman spectra (a) and XRD patterns (b) of samples taken at different times of hydrothermal treatment of aluminosilicate precursor at 90 °C resulting in fully crystalline RHO zeolite after 60 min.

Ex-situ IR and DLS characterization of the amorphous and crystalline RHO zeolite nanoparticles

The average hydrodynamic diameter of the amorphous aluminosilicate and crystalline RHO nanozeolite particles (Figure S 2) were approximately 36 and 120 nm, respectively, as measured by dynamic light scattering (DLS). The polydispersity index (PDI) was 0.2 for the amorphous aluminosilicate and 0.07 for the crystalline RHO. This low PDI value indicates a highly monodisperse sample containing RHO nanoparticles.

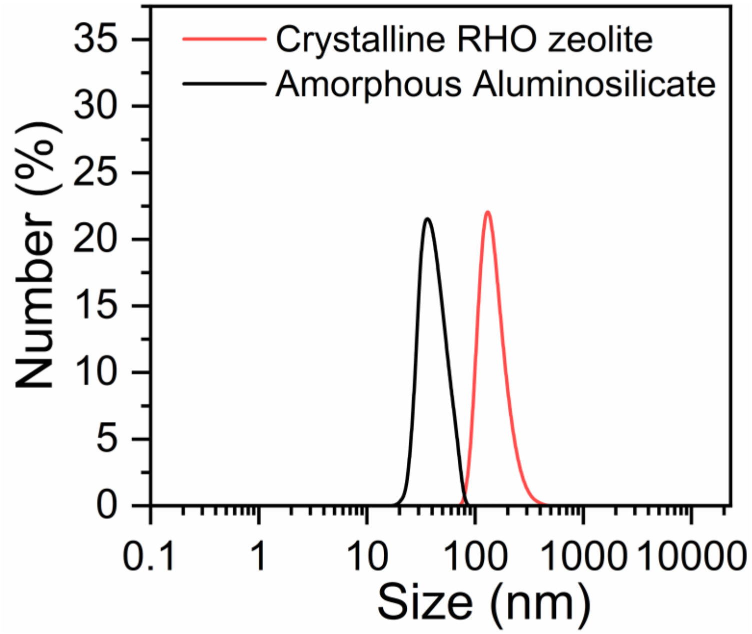

Figure S 2 DLS curves of amorphous aluminosilicate precursor and fully crystalline RHO nanozeolite suspensions prepared at 90 °C for 60 min.

The FTIR spectrum of crystalline RHO sample (Figure S 3) shows broad absorption bands centered at 3441 cm^{-1} and 1633 cm^{-1} attributed to the stretching and bending vibration modes of water molecules adsorbed within the zeolite samples. The most intense bands, observed at 1017 cm^{-1}, corresponds to the vibrations due to the asymmetric stretching of Si–O–Si and Si–O–Al groups, corresponding to the SiO_4 and AlO_4 tetrahedra in the RHO zeolite structure. The absorption band at 763 cm^{-1} is associated with the symmetric stretching vibrations of Si–O–Al–O–T (T = Si or Al), while the band centered at 463 cm^{-1} is attributed to TO_4 (T = Si or Al) tetrahedral bending of zeolite RHO structure.

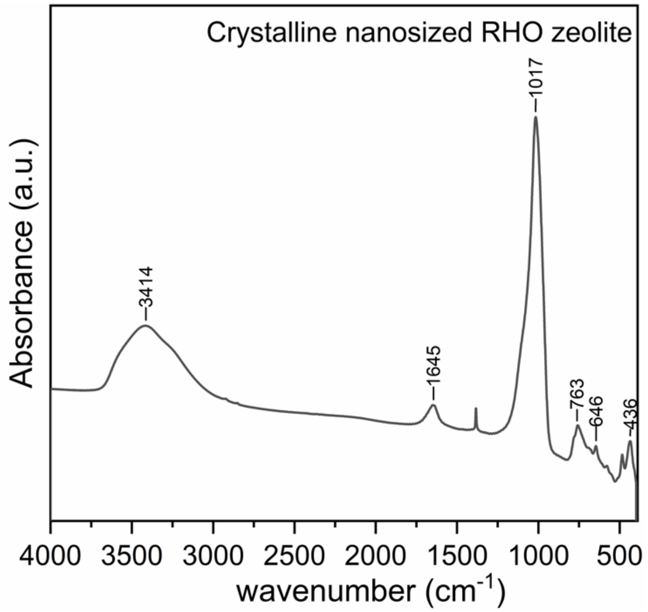

Figure S 3: IR spectra of fully crystalline RHO nanozeolite prepared at 90 °C for 60 min

TEM characterization of the suspension at various electron doses

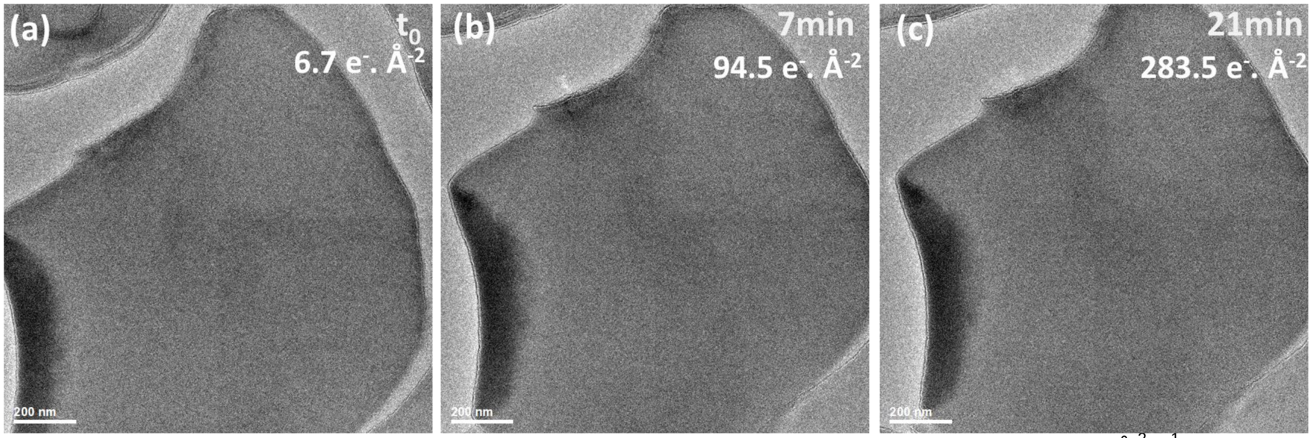

Figure S 4: TEM image sequences of the amorphous matrix acquired at an electron dose rate of 0.22 e^-.Å^{-2}.s^{-1}. The elapsed irradiation time and the calculated cumulative electron dose are shown in bold white in the top right corner of each image.

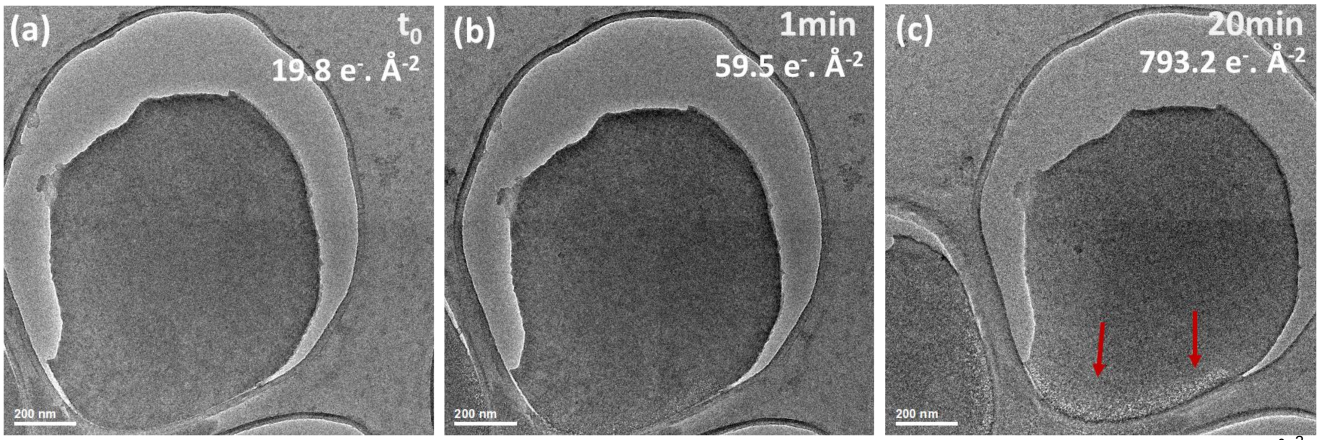

Figure S 5: TEM image sequences of the amorphous aluminosilicate matrix acquired at an electron dose rate of 0. 66 e^-.Å^{-2}.s^{-1}. The elapsed irradiation time and the calculated cumulative electron dose are indicated in bold white in the top right corner of each image. The red cross highlights areas of electron beam damages.

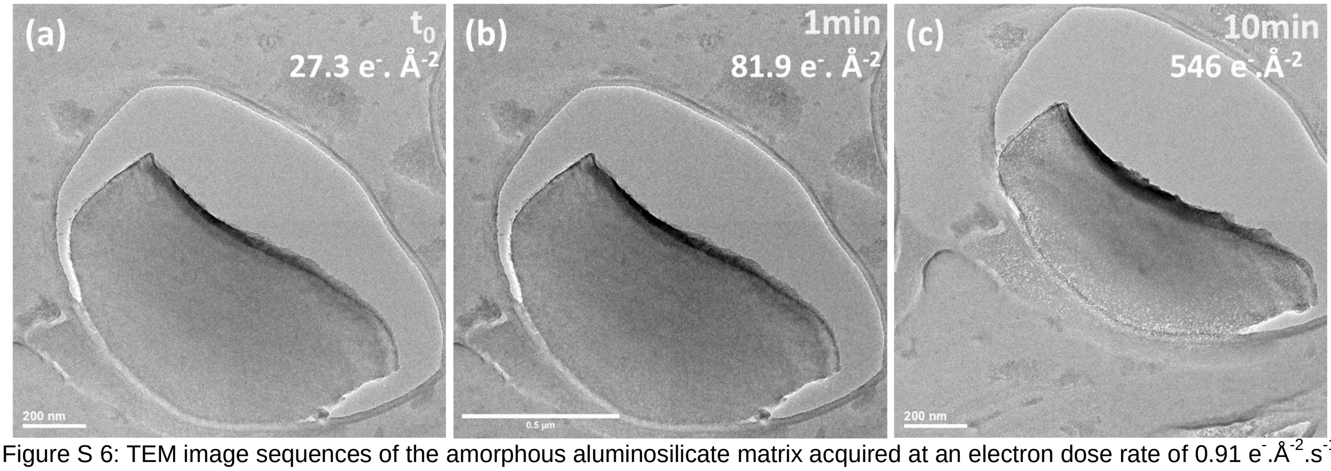

Figure S 6: TEM image sequences of the amorphous aluminosilicate matrix acquired at an electron dose rate of 0.91 e^-.Å^{-2}.s^{-1}. The elapsed irradiation time and the calculated cumulative electron dose are indicated in bold white in the top right corner of each image.

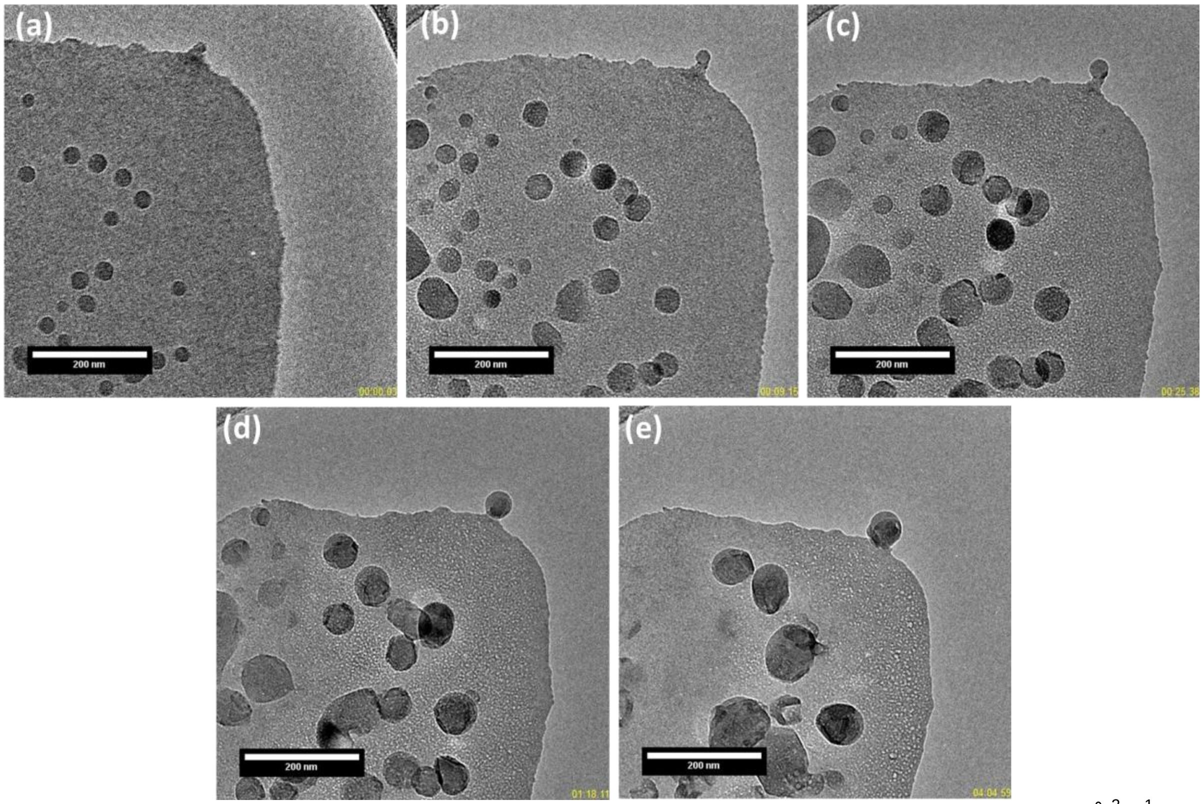

Figure S 7: TEM image sequences of the aluminosilicate matrix acquired at an electron dose rate of 24.6 $e^{-}.Å^{-2}.s^{-1}$ at various times points: (a) 10 s, (b) 19.1 s, (c) 35.4 s, (d) 88.1 s, (e) 254.6 s.

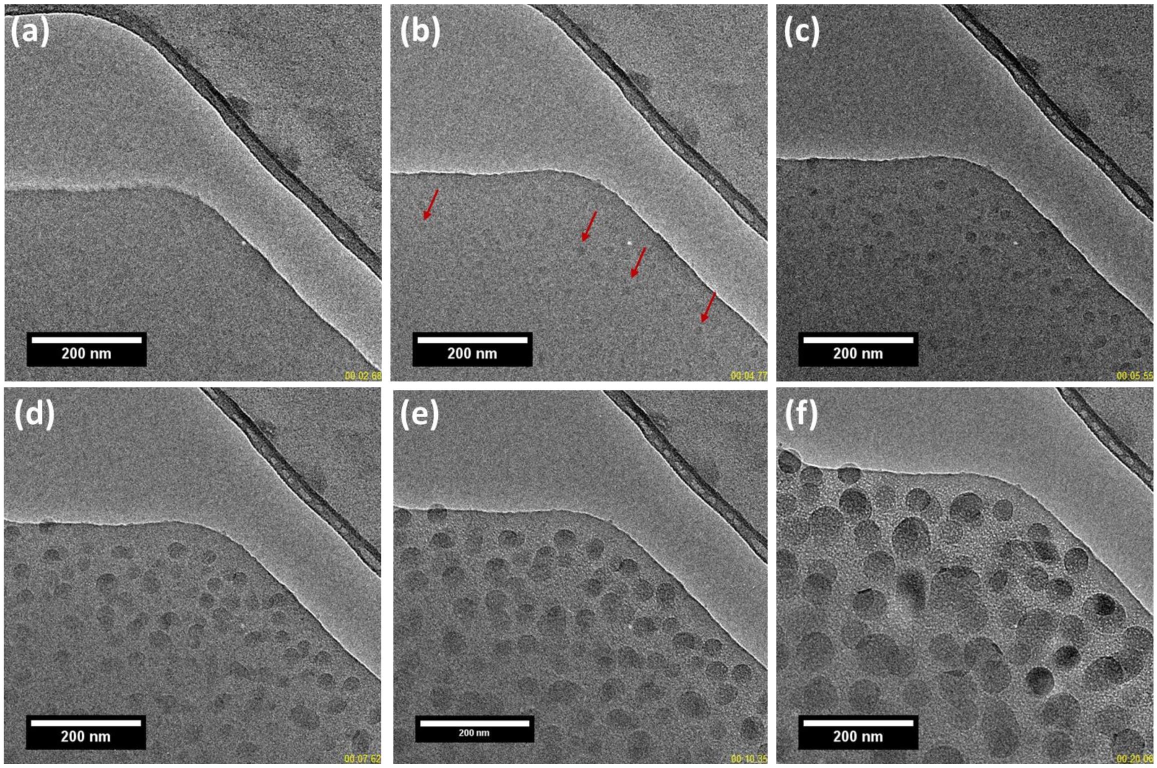

Figure S 8: TEM image sequence of the growth of nanosized RHO zeolites inside the amorphous aluminosilicate matrix at an electron dose rate of 80.7 $e^{-}.Å^{-2}.s^{-1}$. Time points: (a) 0 s, (b) 2.09 s, (c) 2.87 s, (d) 4.94, (e) 7.47 s, (f) 17.32 s. See also Movie S.2.

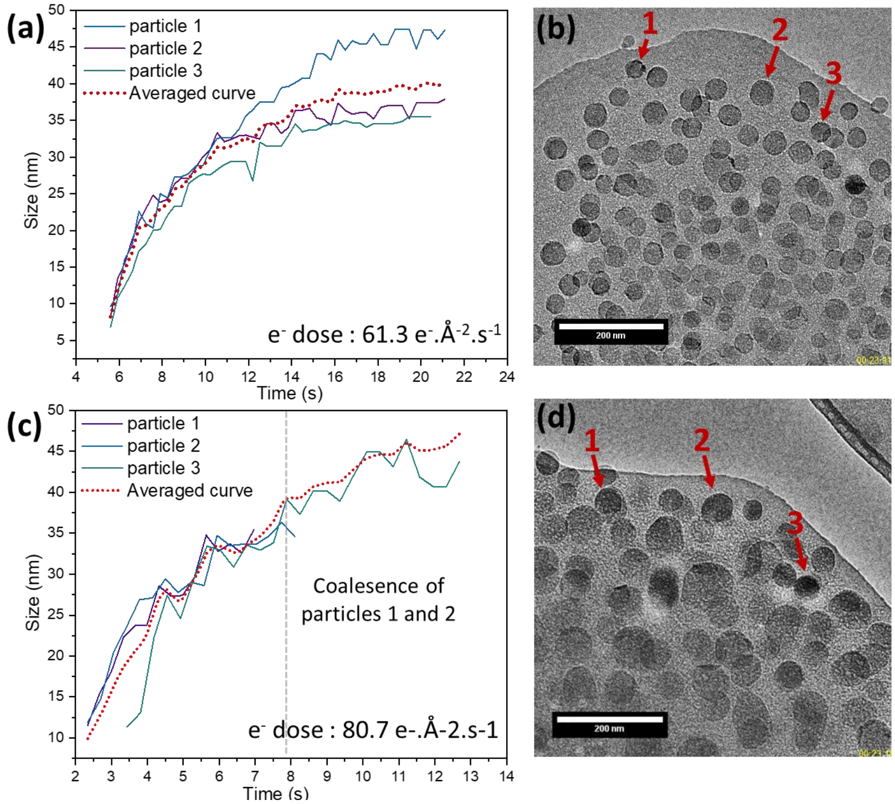

Figure S 9: (a) Size evolution as a function of time for three different particles, indicated in the associated TEM image (b) along with averaged curve (red dotted line) for an electron dose rate of 61.3 e$^-$.Å^{-2}.s^{-1}; (c) Size evolution as a function of time for three particles, indicated in the associated TEM image (d) along with averaged curve (red dotted line) for an electron dose rate of 80.7 e$^-$.Å^{-2}.s^{-1}. Note that the TEM images correspond to the last frame of the growth and were used for the size statistic present in **Error! Reference source not found.**.

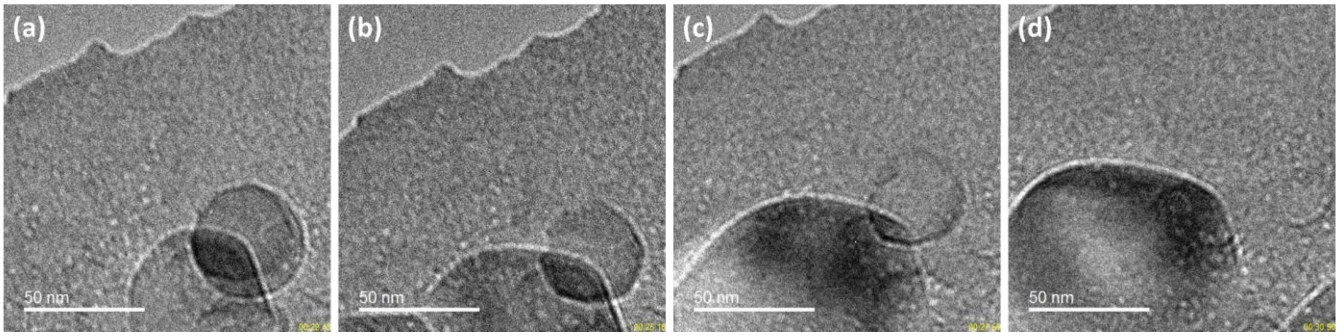

Figure S 10: TEM image sequence showing the dissolution of smaller particles and the growth of larger particle through Ostwald ripening at various times: (a) 1.8 s, (b) 2.9 s, (c) 3.9 s, (d) 5.2 s. Electron dose of 45.9 e$^-$.Å^{-2}.s^{-1}. See also Movie S.6.

Additional Videos

Movie S.1: Dynamic recording of the crystallization of nano-RHO zeolites under the electron beam (20 fps). Video acquired at an electron dose of 24.6 $e^{-}.Å^{-2}.s^{-1}$.

Movie S.2: Dynamic recording of the crystallization of nano-RHO zeolites under the electron beam (6 fps). Video acquired at an electron dose of 61.3 $e^{-}.Å^{-2}.s^{-1}$.

Movie S.3: Dynamic recording of the crystallization of nano-RHO zeolites under the electron beam (15 fps). Video acquired at an electron dose of 80.7 $e^{-}.Å^{-2}.s^{-1}$.

Movie S.4: Dynamic recording of the coalescence and Otswald ripening occurring after the initial homogeneous growth (30 fps). Video acquired at an electron dose of 61.3 $e^{-}.Å^{-2}.s^{-1}$.

Movie S.5: Dynamic recording of the coalescence and Otswald ripening occurring after the initial homogeneous growth (1 fps). Video acquired at an electron dose of 80.7 $e^{-}.Å^{-2}.s^{-1}$.

Movie S.6: Dynamic recording of the growth by Otswald ripening of a single particle at higher magnification (10 fps). Video acquired at an electron dose of 45.9 $e^{-}.Å^{-2}.s^{-1}$.
